# Фиксированные комбинации агонистов рецепторов ГПП-1 и базального инсулина: обзор литературы

**DOI:** 10.14341/probl13312

**Published:** 2024-02-28

**Authors:** Д. В. Куркин, Д. А. Бакулин, Е. И. Морковин, А. В. Стрыгин, Ю. В. Горбунова, Е. В. Волотова, А. И. Робертус, И. Е. Макаренко, В. Б. Сапарова, Р. В. Драй, В. И. Петров

**Affiliations:** Российский университет медицины; Волгоградский государственный медицинский университет; Российский университет медицины; Волгоградский государственный медицинский университет; Волгоградский государственный медицинский университет; Российский университет медицины; Волгоградский государственный медицинский университет; Российский университет медицины; Российский университет медицины; ЗАО «Фарм-Холдинг»; Российский университет медицины; ЗАО «Фарм-Холдинг»; ЗАО «Фарм-Холдинг»; Волгоградский государственный медицинский университет

**Keywords:** инсулинотерапия, сахарный диабет 2 типа, иДегЛира, иГларЛикси, лираглутид, ликсисенатид, арГПП-1

## Abstract

Прогрессирующее течение сахарного диабета 2 типа (СД2) приводит к необходимости назначения инсулинотерапии у значительной части больных. Как правило инсулинотерапия при СД2 сопровождается набором массы тела и существенным увеличением риска гипогликемий. Однако современные опции, такие как фиксированные комбинации агонистов рецепторов глюкагоноподобного пептида-1 (арГПП-1) и базального инсулина, позволяют минимизировать набор веса и риск гипогликемий, а также позволяют большей доле пациентов достигать индивидуальных целей гликемического контроля без ухудшения параметров безопасности. Данный обзор включает описание рандомизированных клинических исследований, а также результатов реальной клинической практики применения двух существующих на сегодняшний день фиксированных комбинаций арГПП-1 и базального инсулина — иДегЛира и иГларЛикси.

## Abstract (en)

The progressive nature of type 2 diabetes mellitus leads to the need for insulin therapy in a significant proportion of patients. Very often start of insulin therapy in type 2 diabetes mellitus (T2DM) is associated with weight gain and a significant increase of hypoglycemia’s risk. However, innovative options, such as fixed ratio combinations of glucagon-like peptide 1 receptor agonists (GLP-1RA) and basal insulin, minimize weight gain and hypoglycemia risks and allow a greater proportion of patients to achieve individual glycemic control goals without compromising safety parameters. This review includes a description of the randomized clinical trials, as well as the results of real clinical practice of the use of two currently existing fixed ration combinations of GLP-1RA and basal insulin — iDegLira and iGlarLixi.

Естественное течение сахарного диабета 2 типа (СД2) характеризуется прогрессирующим снижением функции β-клеток (с потерей примерно 50% на момент постановки диагноза и последующим ухудшением функции примерно на 4–6% в год), что вызывает прогрессирующее же снижение секреции и резерва инсулина [1]. Это явление лежит в основе возникновения потребности в поэтапной интенсификации терапии и назначении инсулинотерапии, при неэффективности таблетированных сахароснижающих препаратов.

В основе текущих рекомендаций по управлению заболеванием лежит достижение целевого уровня гликированного гемоглобина (HbA1c) до 6,5–7,0% или ниже у большинства пациентов при условии, что эти целевые значения достигаются с помощью препаратов, связанных с очень низким риском гипогликемии. Менее строгие целевые значения (<8%) предлагаются для пациентов с тяжелой гипогликемией в анамнезе, ограниченной ожидаемой продолжительностью жизни, выраженными микро- или макрососудистыми осложнениями, сопутствующими или длительно существующими заболеваниями [2].

Для достижения целевого уровня HbA1c рекомендуется использовать последовательное добавление сахароснижающих препаратов, при этом в случае недостижения цели рекомендуется выбирать препараты с высокой сахароснижающей эффективностью, в том числе инсулинсодержащие [2][3]. Несмотря на эти рекомендации, значительная часть пациентов с СД2, включая тех, кто начал лечение инсулином, не достигает целевых значений гликемического контроля. Согласно данным крупного обсервационного исследования, только 28% европейских и американских пациентов с СД2, ранее не получавших инсулин, достигли уровня HbA1c менее 7% через 24 месяца после начала введения базального инсулина (БИ) [4].

Отчасти такие скромные результаты являются следствием проблемы клинической инертности в отношении старта инъекционной терапии. В ретроспективном когортном исследовании 81 573 пациентов с СД2 из Clinical Practice Research Datalink Великобритании с января 2004 по декабрь 2006 гг. было показано, что старт инсулинотерапии откладывают на несколько лет даже при наличии показаний. При этом период от назначения 2–3 таблетированных препаратов до перевода на инсулинотерапию составляет в среднем 6–7 лет [5]. Важнейшими барьерами для своевременной интенсификации терапии являются страх перед увеличением массы тела и высокий риск возникновения гипогликемий. В настоящее время эти факторы можно скорректировать путем назначения современных опций инсулинотерапии (например, фиксированных комбинаций агонистов рецепторов глюкагоноподобного пептида-1 (арГПП-1) и БИ) [6].

Еще одной серьезной проблемой является низкая приверженность к терапии, которая встает особенно остро при возникновении потребности в ее интенсификации у пациентов, не достигших цели при терапии БИ.

По всей видимости, именно фактором приверженности обусловлена колоссальная разница в результатах лечения между рандомизированными клиническими исследованиями (РКИ) и реальной клинической практикой (РКП) применения базисно-болюсной инсулинотерапии (ББИТ) и предварительно смешанных инсулинов. В анализе, сравнившем результаты 8 рандомизированных клинических исследований, в которых изучались ББИТ (n=1893) и готовые смеси инсулинов (n=1517), с данными реальной клинической практики, которые были получены из Британской базы данных пациентов, получавших ББИТ (n=7483) или смешанные инсулины (n=10 744), было показано, что в среднем снижение HbA1c на ББИТ составило -0,33% в РКП, тогда как в РКИ эта разница была: -1,44%. Аналогичная картина была показана и при анализе терапии готовыми смесями инсулинов: согласно полученным результатам HbA1c в РКП в среднем снижался на -0,25%, в РКИ на -1,34% [7].

Интересны также данные интернет-опроса 1250 врачей (600 специалистов, 650 врачей первичного звена), лечащих пациентов с диабетом в Китае, Франции, Японии, Германии, Испании, Турции, Великобритании или США, согласно которым доля врачей, сообщивших, что их пациенты могут успешно самостоятельно корректировать дозы инсулина, составила только 6,2%, при этом только 28,9% специалистов были уверены, что их пациенты вводят БИ каждый день [8].

Таким образом, очевидно, что в РКП далеко не все пациенты с СД2 осваивают сложные схемы инсулинотерапии, что ведет к снижению приверженности и ухудшению клинических результатов лечения. В данном случае уменьшение количества инъекций при комбинировании двух компонентов в одной лекарственной форме может также оказывать положительный эффект на исходы терапии.

## ОБОСНОВАНИЕ ПОДХОДА

Как уже было упомянуто выше, прогрессирующее течение СД2 рано или поздно приводит к необходимости назначения инсулинотерапии. До 2016 года наиболее приемлемым вариантом старта инсулинотерапии считалось назначение БИ, вводимого 1 или 2 раза в день, что зачастую сопровождалось недостаточно выраженной сахароснижающей эффективностью. Для пациентов с HbA1c≥9% вероятность достижения гликемического контроля с помощью одного лишь БИ очень низка, при этом пациент сталкивался с проблемой набора массы тела и увеличения частоты гипогликемий, что снижает приверженность к терапии [9].

Спустя непродолжительное время после внедрения в клиническую практику арГПП-1 стало очевидно, что именно этот класс препаратов может показать наибольшие преимущества при комбинировании с БИ, поскольку прандиальные арГПП-1 влияют на гликемию после еды и способствуют снижению веса, тогда как БИ в основном нацелен на снижение гликемии натощак (табл. 1) [10].

**Table:** Таблица 1. Обоснование рациональности комбинации базального инсулина и арГПП-1 [10]

Аналог базального инсулина	Агонисты рецепторов ГПП-1
Простой режим инициации и подбора дозы Контроль ночной и тощаковой гликемии Меньший риск гипогликемии vs инсулины НПХ Небольшое увеличение массы тела	Простой режим инициации Снижение постпрандиальной гликемии Нет увеличения риска гипогликемии Снижение массы тела

АрГПП-1 усиливают секрецию инсулина и подавляют секрецию глюкагона, способствуя снижению гликемии (инкретиновый эффект), воздействуют на афферентные нейроны блуждающего нерва и/или напрямую на головной мозг, подавляя аппетит и способствуя появлению чувства насыщения. Доказаны выраженные, не связанные с гипогликемическим действием, кардио-, эндотелио- и нейропротективные эффекты арГПП-1, которые реализуются через связь рецептора к ГПП-1 с внутриклеточными сигнальными каскадами, направленными на запуск энергозатратных анаболических процессов [11], а также стимулируют биосинтез инсулина, пролиферацию β-клеток и ингибируют их апоптоз. АрГПП-1 — это класс лекарственных препаратов, назначение которых обеспечивает не только надежный гликемический контроль и снижение массы тела пациентов, но и сопровождается уменьшением риска развития сердечно-сосудистых осложнений СД2. Учитывая физиологическую роль, наличие выраженного гипогликемического действия и широкий спектр ценных плейотропных эффектов (гипофагический, кардио-, эндотелио-, церебро- и панкреопротективный), лекарственная форма (раствор для инъекций) арГПП-1 являются идеальными кандидатами для комбинирования с препаратами БИ для одновременного введения пациентам с СД2.

Преимущества комплексного подхода были показаны в систематическом обзоре 3 рандомизированных клинических исследований (n=1136) сравнения режима арГПП-1 + БИ vs базисно-болюсный режим инсулинотерапии, согласно результатам которого терапия комбинацией арГПП-1 + БИ обеспечивала сравнимый с ББИТ контроль гликемии [12].

Более того, комплементарное действие арГПП-1 и инсулина направлено на различные звенья патогенеза СД2, что потенциально может приводить к реализации широкого спектра плейотропных эффектов и замедлению прогрессии заболевания. Так, например, сегодня известно, что арГПП-1 помимо усиления глюкозозависимой секреции инсулина вызывают усиление чувства насыщения в головном мозге, положительно влияют на функцию миокарда, способствуют замедлению опорожнения желудка, в то время как инсулин способствует ингибированию продукции глюкозы печенью, активации рецепторов инсулина и увеличению утилизации глюкозы в периферических тканях [13][14]. В недавнем исследовательском анализе Ferranini было показано, что переключение с арГПП-1 на комбинацию арГПП-1 и БИ значительно улучшало все показатели, отражающие функцию β-клеток [15].

Таким образом комбинирование инкретинового компонента и БИ в единой лекарственной форме является логичным и патогенетически обоснованным. Именно поэтому сразу две фармацевтические компании создали фиксированные комбинации арГПП-1 и БИ. Ученые компании «Ново Нордиск» объединили в одной инъекции лираглутид 3,6 мг/мл и инсулин деглудек 100 ЕД/мл (иДегЛира), а разработчики компании «Санофи» соединили инсулин гларгин 100 ЕД/мл и ликсисенатид 33 или 50 мкг/мл (иГларЛикси).

## РАНДОМИЗИРОВАННЫЕ КЛИНИЧЕСКИЕ ИССЛЕДОВАНИЯ ФИКСИРОВАННЫХ КОМБИНАЦИЙ (ПРОГРАММА LIXILAN, DUAL)

Обе фиксированные комбинации имеют обширную доказательную базу, подтверждающую их эффективность и безопасность при применении как на старте инсулинотерапии, так и в качестве опции для интенсификации после БИ.

иГларЛикси

Препарат иГларЛикси был изучен в программе исследований под названием LixiLan, а также в исследовании SoliMix.

LixiLan-O

В рандомизированном клиническом исследовании LixiLan-O иГларЛикси сравнивался с базальным инсулином и ликсисенатидом у пациентов с СД2, не достигших цели на терапии пероральными сахароснижающими препаратами (ПССП). Согласно полученным результатам, на терапии иГларЛикси было отмечено более выраженное снижение уровня НbА1с от исходных показателей по сравнению с группами иГлар и Ликси (-1,6 %; -1,3 %; -0,9 % соответственно), уменьшение массы тела на фоне терапии иГларЛикси и Ликси составило 0,3 и 2,3 кг соответственно, в то время как в группе иГлар наблюдалось увеличение массы тела в среднем на 1,1 кг (разница 1,4 кг, р<0,0001). Частота эпизодов документированной симптоматической гипогликемии была сходной в группах иГларЛикси и иГлар (1,4 и 1,2 события/пациенто-год) и меньшей в группе Ликси (0,3 события/пациенто-год). При этом терапия иГларЛикси способствовала улучшению контроля постпрандиальной гликемии по сравнению с иГлар и ассоциировалась с существенно меньшей частотой таких нежелательных явлений, как тошнота (9,6%) и рвота (3,2%), по сравнению с Ликси (24 и 6,4% соответственно). Таким образом, иГларЛикси оказался значительно эффективнее монопрепарата базального аналога инсулина, не уступая по параметрам безопасности в отношении развития гипогликемий, и одновременно показал преимущество по влиянию на массу тела [16].

LixiLan-L

В рандомизированном клиническом исследовании LixiLan-L иГларЛикси сравнивался с увеличением дозы инсулина гларгин 100 ед/мл у пациентов с СД2 с недостаточным гликемическим контролем на ПССП в комбинации с БИ.

При этом среднее снижение HbA1с к 30-й неделе у пациентов, получавших иГларЛикси, составило -1,1%, а у пациентов в группе лечения инсулином гларгин — -0,6%. При этом среднее изменение массы тела в группе иГларЛикси составило -0,7 кг, а у пациентов, получавших инсулин гларгин, — +0,7 кг.

Таким образом, иГларЛикси оказался эффективнее дотитрации инсулина гларгин с преимуществами по влиянию на массу тела [17].

LixiLan-G

В рандомизированном 26-недельном открытом исследовании LixiLan-G изучалась эффективность иГларЛикси у пациентов с СД2, не достигших цели на терапии ПССП в комбинации с арГПП-1. Пациенты были рандомизированы на продолжение текущей терапии или перевод на иГларЛикси. Через 30 недель в группе иГларЛикси среднее снижение HbA1с составило -1,0%, а у пациентов в группе лечения арГПП-1 -0,4%. Среднее значение снижения гликемии через 2 часа после приема пищи на терапии иГларЛикси составило -4,0 ммоль/л по сравнению с -1,1 ммоль/л в группе арГПП-1 [18].

Интересно, что на основании исследования LixiLan-G было проведено несколько очень любопытных дополнительных исследовательских анализов. В работе Stefano DelPrato было показано, что эффективность иГларЛикси не зависит от сохранности резерва функции β-клеток и продолжительности заболевания [19]. В работе E. Ferrannini 2022 г. было показано, что применение иГларЛикси в течение 26 недель приводит к улучшению ряда параметров, отражающих функцию β-клеток, в том числе чувствительности к инсулину, индекса ранней секреции инсулина и коэффициента потенцирования эндогенного инсулина [15].

SoliMix

Исследование SoliMix стало первым прямым рандомизированным контролируемым исследованием, в котором напрямую сравнивались эффективность и безопасность иГларЛикси и двухфазного инсулина аспарт 30 в качестве варианта интенсификации лечения для пациентов с СД2, не достигших целевых значений HbA1c на терапии БИ в сочетании с одним или двумя ПССП. В этом открытом 26-недельном многонациональном исследовании иГларЛикси показал не меньшую сахароснижающую эффективность и даже статистическое превосходство в снижении HbA1c по сравнению с двухфазным аспартом 30. При этом среднее снижение HbA1с к 26-й неделе у пациентов, получавших иГларЛикси, составило -1,3%, а у пациентов в группе лечения двухфазным аспартом 30 — -1,1%. Более того, иГларЛикси продемонстрировал превосходство по влиянию на массу тела (-1,9 кг в сравнении с двухфазным аспартом 30) на 33% меньшую частоту гипогликемий [20]. В исследовании SoliMix также проводился анализ исходов, сообщаемых пациентами по шкалам TRIM-D и GTEE, согласно результатам которого значительно больше пациентов были удовлетворены терапией фиксированной комбинацией (ФК) по сравнению с терапией готовой смесью инсулина [21].

иДегЛира

Препарат иДегЛира был изучен в обширной программе исследований под общим названием DUAL (табл. 2).

**Table:** Таблица 2. Результаты основных исследований программы DUAL [22–29]

	DUAL
Номер исследования	I	II	III	IV	V	VII	VIII
n	1663	413	438	435	557	506	1012
иДегЛира vs.	деглудек/лираглутид	деглудек	лираглутид/эксенатид	плацебо	гларгин	гларгин+аспарт	иГлар
Популяция пациентов с СД2	Инсулин-наивные на ПССП (метформин, пиоглитазон)	БИ+ПССП (метформин, ПСМ или глиниды)	арГПП-1+ПССП	Инсулин-наивные на ПССП (ПСМ+метформин) SU±metformin	Терапия иГлар ≥90 дней до скрининга	Терапия иГлар ≥90 дней до скрининга	Инсулин-наивные на ПССП
начальный HbA1c	8,3%	8,7%	7,8%	7,9%	8,4%	8,2%	8,2%
ΔHbA1c	-1,9% (неделя 26)	-1,9%	-1,3%	-1,5%	-1,8%	-1,5%	-1,9%
Среднее изменение массы тела в группе иДегЛира	-0,5 кг	-2,7 кг	+2,0 кг	+0,5 кг	-1,4 кг	-0,9 кг	+0,5 кг

DUAL I

DUAL I было открытым рандомизированным исследованием III фазы, в котором оценивалась эффективность препарата иДегЛира у пациентов, не достигших цели на ПССП. Через 52 недели среднее снижение HbA1c в группе иДегЛира превосходило таковое в группе инсулина деглудек на 0,46%, а в группе лираглутида — на 0,65% (p<0,001). Кроме того, в группе препарата иДегЛира наблюдалось небольшое снижение массы тела по сравнению со значимым увеличением показателя в группе инсулина деглудек и значимым снижением в группе лираглутида: -0,4; +2,3 и -3,0 кг соответственно [22].

DUAL II

Buse и его коллеги включили в рандомизированное двойное слепое параллельное исследование DUAL II 413 пациентов, не достигших цели на БИ в сочетании с ПССП. В конце 26-недельного исследования среднее изменение HbA1c по сравнению с исходным уровнем составило -1,1% в группе иДегЛира, при этом ФК значимо превосходила по сахароснижающей эффективности инсулин деглудек (р<0,0001). Так как при этом средние суточные дозы инсулина деглудек как в монопрепарате, так и в комбинации с лираглутидом были сопоставимы (в среднем 45 ЕД), это указывает на то, что наблюдаемая разница в результатах лечения была связана с арГПП-1 в составе фиксированной комбинации [23].

DUAL III

В 26-недельном открытом рандомизированном исследовании DUAL III принимали участие пациенты, не достигшие цели на терапии арГПП-1 в сочетании с ПССП, которые были рандомизированы на получение препарата иДегЛира или продолжение текущей терапии арГПП-1 и ПССП.

Согласно результатам исследования, HbA1c снизился в среднем на 1,3% в группе иДегЛира по сравнению с 0,3% у продолжавших терапию арГПП-1 (р<0,001). При этом значительно большее число участников в группе иДегЛира достигли целевых показателей HbA1c<7% и ≤6,5%, чем в группе продолживших прием арГПП-1+ПССП [24].

DUAL IV

В исследование DUAL IV включались взрослые пациенты, получавшие производные сульфонилмочевины в сочетании или без сочетания с метформином. За участниками наблюдали в течение 26 недель, оценивая изменение HbA1c по сравнению с исходным уровнем. HbA1c значимо снизился на 1,5% в группе иДегЛира по сравнению со снижением на 0,5% в группе плацебо (р<0,001) [25].

DUAL V

В клиническом исследовании DUAL V сравнивалась эффективность препарата иДегЛира с продолжением терапии инсулином гларгин у пациентов, не достигших целей гликемического контроля на терапии инсулином гларгин в сочетании с метформином.

Согласно полученным результатам, препарат иДегЛира превосходил инсулин гларгин как по параметрам сахароснижающей эффективности (оцениваемая разница между группами -0,59%, ДИ от -0,74 до -0,45% (р<0,001), так и по влиянию на динамику массы тела -3,2 кг, 95% ДИ от -3,77 до -2,64; (р<0,001) [26].

DUAL VI

В исследовании DUAL VI Харрис и его коллеги изучили влияние режима титрования на общую эффективность препарата иДегЛира. Пациенты с уровнем HbA1c от 7 до 10% на фоне лечения метформином с пиоглитазоном или без него были рандомизированы на терапию препаратом иДегЛира с титрацией один или два раза в неделю. Согласно полученным результатам, между группами не было статистически значимых различий, за исключением изменения массы тела. По завершении 32-недельного исследования пациенты в группе титрования один раз в неделю потеряли в среднем 1 кг по сравнению с потерей 2 кг в группе титрования два раза в неделю, р=0,014 [27].

DUAL VII

DUAL VII стало первым исследованием, в котором препарат иДегЛира сравнили с базисно-болюсным режимом инсулинотерапии. Согласно полученным результатам, препарат иДегЛира не уступал ББИТ по параметрам сахароснижающей эффективности со средним снижением уровня HbA1c на 1,5% в обеих группах. Однако эти результаты были достигнуты при дозе БИ примерно 40 единиц в группе иДегЛира по сравнению с общей суточной дозой инсулина примерно 80 единиц в группе ББИТ [28].

При этом пациенты, получавшие иДегЛира, снизили массу тела, тогда как в группе ББИТ отмечалось значимое ее увеличение (р<0,0001). Таким образом, терапия иДегЛира приводила к снижению HbA1c, сравнимому с таковым на ББИТ, однако при этом с меньшей частотой гипогликемий и снижением массы тела. Все указывает на то, что данная фиксированная комбинация является более привлекательной альтернативой для пациентов, не достигших цели на БИ по сравнению с ББИТ [29][30].

Влияние на время в целевом диапазоне

Особую ценность имеют данные, отражающие вариабельность гликемии на терапии современными инъекционными опциями. В настоящее время такие данные имеются только относительно препарата иДегЛира.

Согласно результатам расширенного периода исследования DUAL I, применение иДегЛира позволяло большему, чем на терапии лираглутидом, количеству пациентов с СД2 поддерживать гликемию в пределах целевых значений. При этом, по данным непрерывного мониторинга глюкозы, препарат иДегЛира обеспечивал значимое снижение колебаний гликемии (р=0,0018) и постпрандиального прироста уровня глюкозы (р=0,0288) по сравнению с препаратом инсулин деглудек [31].

В другом исследовании 24 пациента с СД2 были распределены случайным образом в перекрестном дизайне для получения либо иДегЛира, либо иДегАсп, а затем — иДегАсп или иДегЛира соответственно. Согласно полученным результатам, время в целевом диапазоне было значительно выше на терапии иДегЛира по сравнению с группой, получавшей иДегАсп [32].

## МЕСТО В АЛГОРИТМАХ (ОТЕЧЕСТВЕННЫЕ И ADA/EASD 2022)

В 2023 г. увидели свет новые версии важнейших для каждого практикующего эндокринолога документов — дополненный 11-й выпуск национальных алгоритмов медицинской помощи больных СД и международный консенсус ADA/EASD.

Согласно российскому документу, инициация инсулинотерапии возможна с базального инсулина, готовых смесей инсулина, фиксированных комбинаций базального инсулина и арГПП-1, многократных инъекций инсулина короткого действия (ИКД), инсулина ультракороткого действия (ИУКД) и базального инсулина, для интенсификации после базального инсулина рекомендованы готовые смеси, ФК арГПП-1 и БИ и ББИТ. При этом подчеркивается, что фиксированные комбинации БИ и арГПП-1 по сравнению с применением БИ позволяют большей доле пациентов достигать целевого уровня HbA1c без повышения частоты гипогликемий и увеличения массы тела. Также отмечается, что аналогичные преимущества могут получить пациенты, уже находящиеся на БИ, при переводе на фиксированные комбинации БИ и арГПП-1 по сравнению с переводом на готовые смеси инсулина [2].

В обновленной редакции международного консенсуса ADA/EASD 2022 года ФК арГПП-1 и БИ впервые стали рассматриваться как отдельный класс. При этом указано, что фиксированная комбинация базального инсулина и арГПП-1 обладает большей сахароснижающей эффективностью, чем отдельные компоненты, а кроме того, демонстрирует меньшее увеличение веса и более низкую частоту гипогликемий, чем интенсивные режимы инсулинотерапии, и лучшую желудочно-кишечную переносимость, чем монопрепараты арГПП-1. В основном алгоритме сахароснижающей терапии ФК арГПП-1 и БИ рассматриваются как препараты с очень высокой сахароснижающей эффективностью, наравне с другими инсулинами, инъекционными инкретиномиметиками и комбинациями пероральных сахароснижающих препаратов. В схеме выбора инсулинотерапии рекомендуется рассмотреть назначение ФК арГПП-1 и БИ уже на старте инсулинотерапии. В рекомендациях ADA/EASD 2022 г. также признаются преимущества упрощения лечения с помощью комбинированных препаратов. Отдельно отмечается, что сочетание базального аналога инсулина и арГПП-1 в одной инъекции может быть простым способом уменьшить бремя и сложность лечения [3].

## ДОКАЗАТЕЛЬСТВА, ПОЛУЧЕННЫЕ В РЕАЛЬНОЙ КЛИНИЧЕСКОЙ ПРАКТИКЕ

В последнее время все большее значение придается доказательствам, отражающим результаты лечения в более релевантной популяции и реальных условиях. Ниже представлены результаты применения обеих фиксированных комбинаций из реальной клинической практики.

иГларЛикси

Препарат иГларЛикси активно изучался во время реальной клинической практики по всему миру. Так, эффективность и безопасность его применения рассматривались в итальянском ретроспективном исследовании ENSURE. В данном исследовании были сформированы две группы: в 1-й группе в дополнение к иГларЛикси пациенты получали производные сульфонилмочевины (ПСМ) или инсулин короткого действия, во 2-й группе в сочетании с иГларЛикси пациенты получали метформин и/или ингибиторы натрий-глюкозного котранспортера 2-го типа (иНГЛТ2). Через 6 месяцев терапии в 1-й группе уровень HbA1c снизился на 0,77%, тогда как во 2-й —на 0,92%. Частота гипогликемии была низкой, серьезных гипогликемических событий на терапии иГларЛикси зафиксировано не было [33].

Невероятно интересный вопрос практического характера лег в основу европейского исследования REALI. В данной работе, включившей 1303 пациентов с СД2, получавших терапию иГларЛикси в течение 24 недель, специалисты пытались оценить, насколько время введения препарата влияет на его эффективность. Участники были разделены на четыре подгруппы в зависимости от времени инъекции: перед завтраком (n=436), перед обедом (n=262), перед ужином (n=399) и те, кто изменил время инъекции (n=206). Согласно полученным результатам, численно наибольшее снижение HbA1c наблюдалось у пациентов, применявших препарат до завтрака (1,57%), по сравнению с теми, кто делал это до обеда (1,27%), до ужина (1,42%) или изменявших время инъекции (1,33%). При этом снижение массы тела во всех группах было сопоставимо (на 1,3–2,3 кг). Таким образом, иГларЛикси был эффективен и безопасен независимо от времени применения, однако инъекция перед завтраком способствовала более эффективному гликемическому контролю [34].

Препарат иГларЛикси изучался в условиях реальной клинической практики и в нашей стране. В 2022 г. были опубликованы результаты ретроспективного когортного исследования SOLO, проведенного в г. Москве. В исследование были включены данные электронных медицинских карт 383 взрослых пациентов с СД2 на терапии иГларЛикси. Согласно полученным результатам, терапия иГларЛикси приводила к снижению HbA1c в среднем на 1,74% через 12 месяцев, а также к уменьшению массы тела в среднем на 3,13 кг (p<0,001) без увеличения риска гипогликемий по сравнению с исходным уровнем [35].

В Венгерском исследовании рутинной практики иГларЛикси приводил к среднему снижению HbA1c на -1,6 [ -1,70; -1,41] за 26 недель. При этом эпизоды гипогликемии отмечались у 9,3% пациентов, и во время исследования не было зарегистрировано ни одного эпизода тяжелой гипогликемии [36]. В Румынском мультицентровом (65 исследовательских центров) проспективном неинвазивном открытом исследовании регистра STAR.Ro среднее изменение HbA1c составило -1,3±1,6% за 24 недели, масса тела снизилась на 1,6±4,0. Таким образом, в реальной клинической практике терапия препаратом иГларЛикси в течение 24 недель приводила к значительному снижение HbA1c с меньшим риском гипогликемии и положительным влиянием на массу тела у пациентов с СД2 [37].

В недавно опубликованном исследовании SoliComplex (ретроспективный анализ базы данных обращений за медицинской помощью Optum Clinformatics в США) иГларЛикси сравнивался с ББИТ и готовыми смесями по параметрам приверженности и непрерывности терапии. Согласно полученным результатам, терапия фиксированной комбинацией была ассоциирована со значимо более высокой непрерывностью и численно более высокой приверженностью терапии, а также меньшей частотой гипогликемии, чем применение базисно-болюсной инсулинотерапии при отсутствии увеличения затрат и ресурсов здравоохранения и при этом со сравнимой сахароснижающей эффективностью (по влиянию на HbА1с) [38].

Интересными представляются также данные недавнего многоцентрового обсервационного исследования, включившего 376 пациентов с СД2, не контролируемым на различных режимах инсулинотерапии: готовые смеси (23,2%), базисно-болюсный режим (30,9%) или БИ+ПССП (37,1%), которых переводили на иГларЛикси или иДегЛира за 6 месяцев до сбора данных. Вне зависимости от исходной терапии отмечалось улучшение показателей гликемического контроля с более выраженным влиянием на массу тела препарата иДегЛира. При этом число пациентов с эпизодами гипогликемии уменьшилось с 24 до 7% после перевода на ФК (p<0,001). Комбинированная конечная точка, включающая снижение массы тела и достижение HbA1c ниже 7%, была достигнута у 20,3% пациентов [39].

ИДегЛира

Перевод с ББИТ

В обсервационное, проспективное и нерандомизированное исследование были включены пациенты с СД2, которые были переведены с многократных ежедневных инъекций инсулина на комбинацию деглудек/лираглутид по решению врача. Группу сравнения составили пациенты, продолжавшие получать интенсивную инсулинотерапию. Согласно полученным результатам: HbA1c (8,4 против 7,4%; p<0,0001) и масса тела (94,1 против 93 кг; p<0,0001) были значительно ниже через 6 мес у пациентов, получавших иДегЛира. Улучшение гликемического контроля было достигнуто при применении деглудека/лираглутида, несмотря на снижение общей суточной дозы инсулина [40]. В еще одном ретроспективном когортном Венгерском исследовании терапия иДегЛира в течение 18 мес привела к большему снижению уровня HbA1c и снижению массы тела в сочетании с более низкой частотой гипогликемий по сравнению с интенсивной инсулинотерапией у пациентов с СД2 [41].

Как показали результаты ретроспективного обсервационного исследования, проведенного в Перудже с февраля 2018-го по апрель 2019 г., иДегЛира продемонстрировал эффективность в реальной клинической практике в снижении уровня HbA1c у самых разных категорий пациентов: не достигших цели на базальном инсулине + ПССП, на арГПП-1 и интенсивных режимах инсулинотерапии. Полученные данные свидетельствуют о том, что в реальной практике переход на иДегЛира является альтернативным вариантом для пациентов с неудовлетворительным гликемическим контролем или с побочными эффектами, такими как увеличение массы тела и гипогликемия, от других видов инсулинотерапии [42].

Результаты реальной клинической практики применения иДегЛира в США продемонстрировали, что данная опция терапии приводит к снижению HbA1c в среднем на -0,8% у тех, кто ранее получал БИ, до -1,0% для тех, кто ранее не получал инъекционную терапию (p<0,0001 для всех) [43].

## ЗАКЛЮЧЕНИЕ

Таким образом, в реальной практике перевод на фиксированную комбинацию арГПП-1 и базального инсулина у пациентов с длительно неадекватно контролируемым СД2 на различных схемах инсулинотерапии может быть эффективной альтернативой сложным режимам в достижении целей гликемического контроля, но при этом с более благоприятным профилем безопасности, повышая приверженность и улучшая клинические результаты лечения. Кроме того, уменьшение количества инъекций при комбинировании двух компонентов в одной лекарственной форме может также оказывать положительный эффект на исходы терапии, а сочетанное действие арГПП-1 и инсулина патогенетической терапии СД2 приводит к реализации широкого спектра плейотропных эффектов и замедлению прогрессии заболевания.

## ДОПОЛНИТЕЛЬНАЯ ИНФОРМАЦИЯ

Источники финансирования. Работа выполнена по инициативе авторов без привлечения финансирования.

Конфликт интересов. Авторы декларируют отсутствие явных и потенциальных конфликтов интересов, связанных с содержанием настоящей статьи.

Участие авторов. Д.В. Куркин — идея и планирование структуры работы; А.И. Робертус, Д.А. Бакулин, Ю.В. Горбунова, В.Б. Сапарова — сбор материала и написание черновика рукописи; Е.И. Морковин, А.В. Стрыгин, Е.В. Волотова — редактирование и утверждение финальной версии рукописи; И.Е. Макаренко, Р.В. Драй, В.И. Петров — написание материала по узкоспециализированным разделам, утверждение финальной версии рукописи.

Все авторы одобрили финальную версию статьи перед публикацией, выразили согласие нести ответственность за все аспекты работы, подразумевающую надлежащее изучение и решение вопросов, связанных с точностью или добросовестностью любой части работы.
